# The integrated stress response induces R-loops and hinders replication fork progression

**DOI:** 10.1038/s41419-020-2727-2

**Published:** 2020-07-16

**Authors:** Josephine Ann Mun Yee Choo, Denise Schlösser, Valentina Manzini, Anna Magerhans, Matthias Dobbelstein

**Affiliations:** Institute of Molecular Oncology, Göttingen Center of Molecular Biosciences (GZMB), University Medical Center Göttingen, 37077 Göttingen, Germany

**Keywords:** DNA, Tumour-suppressor proteins

## Abstract

The integrated stress response (ISR) allows cells to rapidly shutdown most of their protein synthesis in response to protein misfolding, amino acid deficiency, or virus infection. These stresses trigger the phosphorylation of the translation initiation factor eIF2alpha, which prevents the initiation of translation. Here we show that triggering the ISR drastically reduces the progression of DNA replication forks within 1 h, thus flanking the shutdown of protein synthesis with immediate inhibition of DNA synthesis. DNA replication is restored by compounds that inhibit eIF2alpha kinases or re-activate eIF2alpha. Mechanistically, the translational shutdown blocks histone synthesis, promoting the formation of DNA:RNA hybrids (R-loops), which interfere with DNA replication. R-loops accumulate upon histone depletion. Conversely, histone overexpression or R-loop removal by RNaseH1 each restores DNA replication in the context of ISR and histone depletion. In conclusion, the ISR rapidly stalls DNA synthesis through histone deficiency and R-loop formation. We propose that this shutdown mechanism prevents potentially detrimental DNA replication in the face of cellular stresses.

## Introduction

The integrated stress response (ISR) is widely known as a mechanism to shutdown the synthesis of most proteins when the cell suffers various stresses^1^ through the activation of the following kinases. Protein kinase R (PKR) is activated upon virus infection and accumulation of double-stranded RNA. PKR-like endoplasmic reticulum kinase (PERK) becomes active when unfolded proteins accumulate in the endoplasmic reticulum. General control nonderepressible 2 (GCN2) responds to amino acid deprivation. And heme-regulated inhibitor (HRI) is triggered in the case of heme depletion in erythrocytes. Each of these kinases triggers the phosphorylation of the alpha subunit of translation initiation factor eIF2 at Serine 51^2^. This modification of eIF2 shuts down the translation of most mRNAs, with the exception of a few mRNAs that employ alternative mechanisms of translation initiation. One of these exceptions is the transcription factor ATF4, which is synthesized with greater efficiency as part of the ISR^3,4^ and then triggers a transcriptional program to counteract the specific stress stimuli^5^. The ISR thus prevents further damage to the cell by avoiding further protein synthesis in the context of proteotoxic stress, or as part of a defense mechanism against virus infection or nutrient depletion.

Besides gene expression, the replication of DNA represents an extreme demand on the cell with regard to metabolic activity and energy consumption. For one round of DNA replication, each human cell must synthesize and incorporate 2 × 3 × 10^9^ dNTPs. This raises the question whether the ISR might also affect the replication of DNA, perhaps protecting the cell in the context of nutrient deprivation or infection. And indeed, the replication of DNA is a highly regulated process. Regulation is not only implied by the control of cell cycle progression. Rather, even during S phase, the cell can stall the progression of replication forks^6^. One example of the underlying mechanisms is provided by the kinase MAPKAPK2, the activation of which diminishes replication fork progression^7,8^. Also, the absence of the tumor suppressor p53 or its target gene product Mdm2 can each enhance replication stress^9,10^. Another way of slowing down DNA replication consists in the lack of histone supply, e.g., by depleting histone chaperones^11,12^. In this situation, the newly synthesized DNA can no longer associate with nucleosomes to a sufficient extent. By mechanisms that are currently not fully explained, this leads to a reduction in DNA synthesis^11–13^. Finally, replication stress can be induced by the formation of R-loops, i.e., DNA:RNA hybrids that form by the looping out of the non-template strand of DNA after transcription, allowing the newly synthesized RNA to rehybridize with its template strand^14,15^. Such R-loops represent obstacles to DNA replication^16–20^.

Previous findings provided hints that the ISR might not only affect the synthesis of proteins but also that of DNA^21,22^, with the earlier report mainly focusing on the drug thapsigargin and its role in replication through interfering with calcium homeostasis. On the other hand, Cabrera et al.^22^, uses thapsigargin to hinder proper protein folding (induce “ER stress”), which subsequently impaired firing of origins and hence overall DNA synthesis. The mechanism was suggested to occur through the activation of claspin and its associated kinase Chk1^22^. Moreover, cycloheximide, a compound that inhibits overall protein synthesis, was found to diminish histone synthesis and slow down DNA replication^12,23^. This raises the question whether the ISR might generally interfere with DNA replication progression, through a shortage of histone synthesis.

Here we show that the ISR triggered by various kinases each interferes with the progression of DNA replication forks in U2OS cells. This can be mimicked by the depletion of histones. Strikingly, the removal of R-loops by RNaseH1, or the overexpression of histones, restores DNA replication upon ISR. In addition, histone depletion alone led to an accumulation of R-loops. This suggests a general mechanism that links ISR to the impairment of replication forks, apparently through histone depletion and R-loops.

## Materials and methods

### Lead contact and materials availability

Further information and requests for resources and reagents should be directed to and will be fulfilled by the lead contact Matthias Dobbelstein (mdobbel@uni-goettingen.de).

This study did not generate unique reagents.

### Experimental model and subject details

#### Cell culture

The human osteosarcoma cell line U2OS (p53 proficient, female) was purchased from ATCC (RRID:CVCL_0042). Cells were maintained in Dulbecco’s modified Eagle’s medium (DMEM) supplemented with 10% fetal bovine serum (Merck), 2 mM l-glutamine (Life Technologies), 50 units/ml penicillin, 50 μg/ml streptomycin (Gibco), and 10 µg/ml Ciprofloxacin (Bayer) at 37 °C in a humidified atmosphere with 5% CO_2_. Cells used were routinely tested and ensured to be negative for mycoplasma contamination.

### Method details

#### Treatments and transfections

Cells were treated with thapsigargin (Thap, Sigma), 1H-Benzimidazole-1-ethanol, 2,3-dihydro-2-imino-alpha-(phenoxymethyl)-3-(phenylmethyl)- monohydrochloride (BEPP, Sigma), l-Histidinol (l-Hist, Sigma), (E)-2-(2-Chlorobenzylidene) hydrazinecarboximidamide (Sephin, Sigma), *trans*-*N*,*N*′-(Cyclohexane-1,4-diyl)bis(2-(4-chlorophenoxy)) acetamide (Integrated stress response inhibitor or ISRIB, Sigma), GSK2606414 (PERK inhibitor or PERK i, Calbiochem), gemcitabine (Gem, Actavis), Cycloheximide (CHX, Sigma), 5,6-Dichloro-1-β-d-ribofuranosylbenzimidazole (DRB, Sigma), or LDC067 (Selleckchem) as indicated in the figure legends. Thap, BEPP, Sephin, ISRIB, PERK i, DRB, and LDC067 were dissolved in DMSO, l-Hist, and gemcitabine dissolved in water, and CHX was dissolved in 100% ethanol.

siRNA transfections were performed using Lipofectamine 3000 (Life Technologies). Cells were reverse transfected with 100 nM siRNA against SLBP (Ambion, custom made, pool of 3 siRNAs) or negative control scrambled siRNA (Ambion, pool of 2 siRNAs), medium replenished after 24 h and cells harvested 40 h post-transfection. For plasmid overexpression, 2 µg of the respective plasmids were forward transfected using Lipofectamine 2000. Medium was replenished after 6 h, and cells were harvested for experiments 24 h post-transfection. The following plasmids were used.

**Table:** 

Plasmid	Origin
pICE-NLS-mCherry	Addgene #60364
pICE-RNaseH1-NLS-mCherry	Addgene #60365
pICE-RNaseH1-D10R-E48R-NLS-mCherry	Addgene #60367
pFRT-ToDest-FlagHA	Addgene #26361
pFRT-ToDest-FlagHA-RNaseH1	Addgene #65782
pCDNA3.1-Flag-H2A	Addgene #63560

#### Cell synchronization

To obtain a majority population of cells in S phase, cells were synchronized using double thymidine block. Briefly, cells were seeded accordingly and allowed to settle and attach onto plates or coverslips for at least 6 h, then treated with 2 mM thymidine (Sigma). After 16 h, cells were washed once in PBS and then replenished with fresh DMEM for 8 h prior to the second thymidine block (2 mM) for another 16 h. Depending on the assay, cells were released into fresh DMEM for 1 h (celigo proliferation assay) or 4 h (R-loop detection on cells treated with CHX) prior to treatment, harvest and analysis.

#### Immunoblot analysis

Cells were washed once in PBS and harvested in radioimmunoprecipitation assay (RIPA) lysis buffer (20 mM TRIS-HCl pH 7.5, 150 mM NaCl, 10 mM EDTA, 1% Triton-X 100, 1% deoxycholate salt, 0.1% SDS, 2 M urea) in the presence of protease inhibitors. Samples were briefly sonicated to disrupt DNA-protein complexes. The protein extracts were quantified using the Pierce BCA Protein assay kit (ThermoScientific Fisher). Protein samples were boiled at 95 °C in Laemmli buffer for 5 min, and equal amounts were analyzed by sodium dodecyl sulfate polyacrylamide gel electrophoresis (SDS-PAGE). Subsequently, proteins were transferred onto a nitrocellulose membrane, blocked in 5% (w/v) non-fat milk in PBS containing 0.1% Tween-20 for 1 h and incubated with primary antibodies at 4 °C overnight followed by incubation with peroxidase-conjugated secondary antibodies (donkey anti-rabbit or donkey anti-mouse IgG, Jackson Immunoresearch). The proteins were detected using either Super Signal West Femto Maximum Sensitivity Substrate (ThermoFisher) or Immobilion Western Substrate (Millipore).

Soluble histones were extracted as described^12^. Briefly, cells were washed once in PBS and harvested in a low detergent, hypotonic buffer (10 mM Tris, pH 7.4, 2.5 mM MgCl_2_, and 0.5% NP-40) for 10 min on ice. Following centrifugation at 1000×*g*, the concentration of the solubilized proteins was determined as described above and equal amounts were analyzed by SDS-PAGE.

**Table:** 

Antibodies	Source (catalog number)	Research resource identifiers (RRID)
ATF4 (D4B8)	Cell Signaling (#11815)	RRID:AB_2616025
Chk1	Cell Signaling (#2360)	RRID:AB_2080320
eIF2alpha	Cell Signaling (#9722)	RRID:AB_2230924
Flag	Sigma (F1804)	RRID:AB_262044
gamma H2AX, γH2AX (S139)	Cell Signaling (#2577)	RRID:AB_2118010
H3	Abcam (ab1791)	RRID:AB_302613
H3K56ac	Cell Signaling (#4243)	RRID:AB_10548193
H4K5ac (EP1000Y)	Abcam (ab51997)	RRID:AB_2264109
H4K12ac (EPR17906)	Abcam (ab177793)	RRID:AB_2651187
HSC70	Santa Cruz (sc-7298)	RRID:AB_627761
mCherry	Abcam (ab167453)	RRID:AB_2571870
phospho-Chk1 (S317)	Cell Signaling (#2344)	RRID:AB_331488
phospho-eIF2alpha (S51)	Cell Signaling (#9721)	RRID:AB_330951
RNaseH1	Abcam (ab56560)	RRID:AB_945244
SLBP (EPR12673)	Abcam (ab181972)	N/A

#### DNA fiber assay

DNA fiber assays were performed as described previously^9^. Briefly, cells were incubated with 5-chloro-2′-deoxyuridine (CldU, Sigma-Aldrich) for 30 min, followed by 60 min incubation with 5-iodo-2′-deoxyuridine (IdU, Sigma-Aldrich) in the presence of inhibitors or treatments as indicated. For the 7-label assay, cells were incubated with CldU for 1 h and then pulsed labeled with IdU and CldU for 15 min each for a total duration of 1.5 h.

Cells were lysed using spreading buffer (200 mM Tris pH 7.4, 50 mM EDTA, 0.5% SDS) and DNA fiber spread on glass slides prior to fixation in a methanol:acetic acid solution (3:1). Upon treatment with 2.5 M HCl, fibers were incubated with rat anti-BrdU antibody (Abcam, RRID:AB_305426, 1:1000, to detect CldU) and mouse anti-BrdU (Becton Dickinson, RRID:AB_10015219, 1:400, to detect IdU) for 1 h at room temperature, then fixed with 4% paraformaldehyde in PBS for 10 min. Slides were incubated with Alexa Fluor 555-conjugated goat anti-rat IgG antibody (RRID:AB_141733) and Alexa Fluor 488-conjugated goat anti-mouse IgG antibody (RRID:AB_138404) (both from ThermoFisher, 1:200) for 2 h at room temperature.

#### S9.6 immunofluorescence

Cells were seeded on glass coverslips, transfected or treated with reagents accordingly and fixed with 4% paraformaldehyde in PBS for 10 min. Then, cells were permeabilized with 0.5% Triton-X 100 in PBS for 15 min, blocked with 3% bovine serum albumin (BSA) in PBS containing 0.1% Tween-20 for 1 h and incubated overnight at 4 °C with S9.6 antibody (Kerafast, RRID:AB_2687463, 1:100, to detect DNA:RNA hybrids). Coverslips were washed in PBS prior to incubation with Alexa Fluor 488-conjugated donkey anti-mouse IgG antibody (ThermoFisher, RRID:AB_141607, 1:250) for 2 h and subsequently counterstained with 0.5 µg/ml DAPI (Sigma) for 5 min prior to mounting using the Fluorescent Mounting Medium from DakoCytomation (#S302380-2) and imaged.

#### Dot blot analysis

Dot blots were conducted as described previously^24^. Cells were seeded, treated with Thap, BEPP or CHX as indicated and harvested. Prior to CHX treatment, cells were synchronized using double thymidine block as described (chapter “Cell synchronization”) and released into fresh DMEM for 4 h prior to addition of CHX. Cells were washed once in PBS and fixed with 1.1% paraformaldehyde in a solution of 0.1 M NaCl, 1 mM EDTA, 0.5 mM EGTA, and 50 mM HEPES pH 7 for 30 min at room temperature. To quench the cross-linking reaction, glycin was added to a final concentration of 0.125 M for 5 min. Subsequently, the cells were lysed in 1% Triton-X 100, 0.15 M NaCl, 1 mM EDTA, 0.3% SDS with protease inhibitors. The cell lysates were sonicated for 10 cycles (30 s on/off) (Bioruptor, Diagenode) and then subjected to 2 mg/ml proteinase K (ThermoFisher) treatment for 1 h at 50 °C. DNA was isolated using phenol-chloroform extraction and DNA concentration normalized between samples.

The DNA (1.3 µl) was spotted onto pre-wet nitrocellulose membrane, allowed to air dry and then cross-linked with UVC for 5 min. The membrane was blocked in 5% BSA in PBS containing 0.25% Tween-20 for 30 min at room temperature and subsequently incubated with S9.6 antibody (Kerafast, 1:300) in blocking solution overnight at 4 °C. Following incubation with peroxidase-conjugated donkey anti-mouse IgG (Jackson Immunoresearch, RRID:AB_2340773, 1:10,000), DNA:RNA hybrids (as measured using S9.6 intensity) were detected using Super Signal West Femto Maximum Sensitivity Substrate (ThermoFisher). To confirm the specificity of the antibody, one half of the DNA samples were also pre-treated with RNaseH (0.03 U/ng DNA, Ambion ThermoFisher) for 3 h at 37 °C prior to spotting. As a loading control, the membrane was subsequently incubated with antibodies to single-stranded DNA (ssDNA). Briefly, the membrane was incubated with 2.5 M HCl for 15 min (to denature the DNA), washed with PBS, and incubated with antibody to ssDNA (Millipore, RRID:AB_570342, 1:1000) for 2 h at room temperature. The detection of ssDNA was performed following exposure to secondary antibody using Super Signal West Femto Maximum Sensitivity Substrate (ThermoFisher).

#### EdU incorporation assay

5-ethynyl-2′-deoxyuridine (EdU, ThermoFisher Scientific, #A10044) was added to exponentially growing cells to a final concentration of 20 µM for 1 h until harvest. Prior to imaging, the cells were fixed and permeabilized as done for immunofluorescence staining. The following reagents were added to 100 mM Na-Phosphate buffer (pH 7) in the following order: 5 µM Alexa Fluor 488 picolyl-azide or 5 µM Alexa Fluor 594 picolyl-azide (Jena Biosciences, #CLK-1276-1 or #CLK-1296-1), 100 µM CuSO_4_ (Jena Biosciences, #CLK-MI004) in 500 µM tris-hydroxypropyltriazolylmethylamine (THPTA; Sigma-Aldrich, #762342) and 5 mM Na-Ascorbate (Jena Biosciences, #CLK-MI005). The click reaction was performed for 1 h on a shaker, at room temperature and protected from light. Samples were subsequently washed thrice for 10 min with PBS, followed by incubation with 0.3 µg/ml DAPI (Sigma-Aldrich, #D9542) for 10 min.

#### Proliferation assay (Celigo)

To study the long-term effect of ISR on cells in S phase, proliferation assay was conducted on synchronized cells. Cells were seeded in technical duplicates in 24-well plates, synchronized using double thymidine block (as described), and released into fresh medium for 1 h then treated with BEPP (30 µM) for 6 h to ensure ISR activation during S phase of the cells. During synchronization, cells were also transfected with plasmids to RNaseH1 or an empty vector control as described previously. After 6 h of treatment, medium was replenished and confluency of cells at day 0 was measured using Celigo Imaging Cytometer (Nexcelom Bioscience). Measurements were made subsequently every 24 or 48 h and medium was changed prior to every measurement.

### Quantification and statistical analysis

#### DNA fiber analysis

To avoid bias, data acquisition and analysis were conducted in a double-blinded manner where identities of the samples were blinded prior to imaging and analysis. Whenever possible, a minimum of 100 DNA fiber structures^25^ were visualized with fluorescence microscopy (Axio Scope A1 microscope (Zeiss) equipped with an Axio Cam MRc/503 camera) and analyzed.

For the 7-label fiber assay, the number of labels incorporated was counted using the cell counter plugin on Fiji. Fork stalling was then calculated by dividing the number of tracks with less than all seven labels by the total number of tracks and converted into percentage. The length of the second to third label was measured to determine the replication progression for the 7-label fiber assay. The Fiji software (RRID:SCR_002285)^26^ was used to measure the labeled tracks in pixels and converted to micrometers using the conversion factor of 1 µm = 5.7 pixels (as determined by measuring scale bar under the same microscope settings) and then to kilo base (kb) using the conversion factor 1 µm = 2.59 kb. Rate of fork progression was calculated by dividing the number of bases by the labeling time of the track.

For the 2-label fiber assays, fibers were analyzed for their IdU track length and IdU fork progression rate calculated as described.

A summary of the fiber assay data containing information on the number of fibers sampled, mean, median, and standard deviation of each condition can be found in Supplementary Table 1.

The raw data of each fiber assay showing the analysis conducted (as described above) can be found in Supplementary Table 2.

#### Nuclear quantification of immunofluorescence

Images were acquired (same exposure time for all images for each fluorescent channel per experiment) with Axio Scope A1 microscope (Zeiss) equipped with an Axio Cam MRc/503 camera.

The Fiji software was used for automated analysis and quantification of nuclear S9.6 or EdU staining. DAPI staining was used to identify regions of interest (nuclei) prior to measuring mean intensity of the Alexa Fluor 488 staining (S9.6), Alexa Flour 488 picolyl-azide or Alexa Fluor 594 picolyl-azide (EdU). At least 200 cells were subjected to analysis and quantification.

#### Statistical testing

Statistical testing was performed using Graph Pad Prism 6 (RRID:SCR_002798). For fiber assay and immunofluorescence experiments where normally distributed data cannot be assumed, Mann–Whitney *U* test was used to calculate significance. For the other experiments, a two-sided unpaired Student’s *t*-test was calculated. Significance was assumed where *p*-values ≤ 0.05. Asterisks represent significance in the following way: ****p ≤ 0.0001, ***p ≤ 0.005; **p ≤ 0.01; *p ≤ 0.05.

## Results

### DNA replication is compromised shortly after ISR induction

**Fig. 1:**
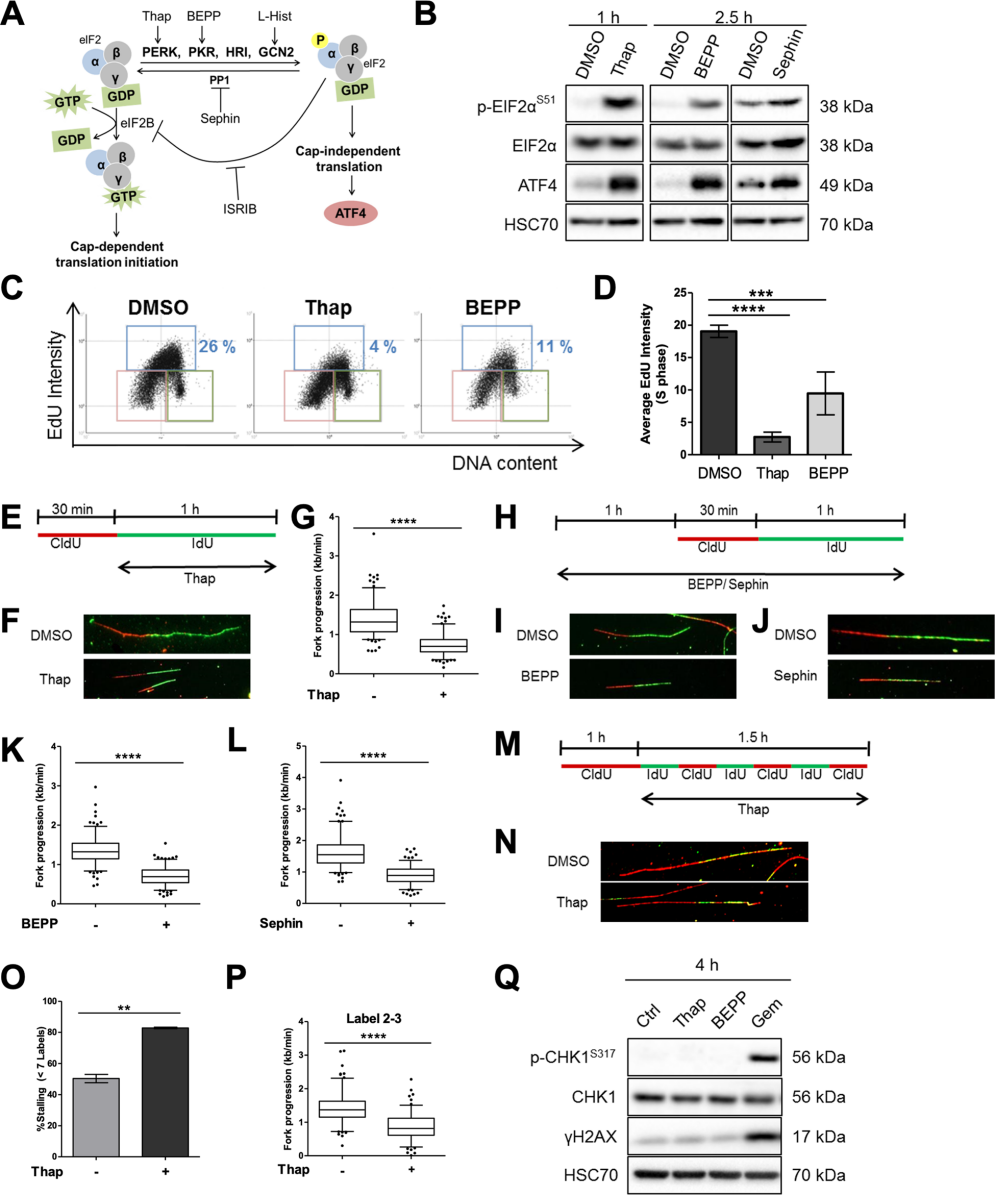
DNA replication is compromised shortly after ISR induction. **a** Schematic representation of the ISR that can be activated upon stimulation of the kinases PERK, PKR, or GCN2 or upon inhibition of the phosphatase PP1 using thapsigargin, BEPP-monohydrochloride, l-Histidinol or Sephin, respectively. Activation of ISR can be measured by an increase in eIF2alpha phosphorylation or by the accumulation of ATF4. ISR can be inhibited using a small molecule inhibitor, ISRIB. **b** Immunoblot analysis of cells treated with Thap (4 μM), BEPP (10 μM), or Sephin (25 μM) to confirm ISR induction. HSC70 as loading control. **c** Representative horseshoe plots showing EdU incorporation in relation to DNA content (DAPI) of cells treated with DMSO, Thap (4 μM, 1 h) or BEPP (10 μM, 2.5 h). The different gates are highlighted as follow: G1 (pink), S (blue), G2/M (green). The percentage of S phase cells is indicated for the respective treatments. **d** Average EdU staining intensity of cells in S phase as determined from the plots in **c** and displayed as mean ± SD. For second replicate, see Supplementary Fig. S1B. **e** U2OS cells were incubated with 5′-chloro-2′-deoxyuridine (25 μM CldU, 30 min) followed by 5-iodo-2′-deoxyuridine (250 μM IdU, 60 min) in the presence of 4 μM Thap prior to harvesting for DNA fiber analysis. **f** Representative labeled tracks of newly synthesized DNA incorporating CldU (red) and IdU (green) of cells in **e**. **g** Fork progression as determined from IdU track length (kb/min), displayed as 5–95 percentile whiskers box plot of Thap-treated cells. Box plots represent data from one out of three independent experiments. See Supplementary Fig. S1C, D for additional experiments. **h** U2OS cells were pre-treated with 10 μM BEPP or 25 μM Sephin for 1 h and subsequently incubated with CldU (30 min) and IdU (60 min) in the presence of these reagents and then harvested for analysis. **i**, **j** Representative fiber tracks as visualized by immunostaining of CldU (red) and IdU (green) of BEPP (**i**) or Sephin (**j**)-treated cells. **k**, **l** Fork progression calculated from the IdU label (kb/min) of BEPP (**k**) or Sephin (**l**)-treated cells. Fork progression displayed as boxplots with 5–95 percentile whiskers, which are representative of one out three independent experiments. See Supplementary Fig. S1E–H. **m** Cells were pulsed labeled with CldU (25 μM, 60 min) and then alternately with IdU (25 μM) and CldU (25 μM) for 15 min intervals for a duration of 1.5 h in the presence of Thap (4 μM), then harvested for 7-label fiber assay analysis^9^. From this, the number of labels incorporated was used for fork stalling analysis and the length of labels 2–3 was used for fork progression analysis. **n** Representative images of fiber tracks that have incorporated 7 labels. **o** Percentage of forks with less than 7 labels indicating higher fork stalling rate of cells treated with Thap. Chart represents mean ± SD of two independent experiments. **p** Velocity of fork determined from track length of labels 2 to 3 displayed as box plots (5–95 percentile whiskers). Plot is a representative of two independent experiments. See Supplementary Fig. S1M. **q** Cells were treated with Thap (4 μM), BEPP (10 μM) or Gem (500 nM) for 4 h and then harvested for western blot analysis. DNA damage signaling was evaluated through Chk1 phosphorylation and gamma H2AX induction. Total Chk1 levels and HSC70 were used as loading controls. Gemcitabine treatment was included as a positive control.

The ISR triggers a shutdown of protein synthesis, representing an emergency response to nutrient deprivation or proteotoxic stress. Here, we tested whether this response might also affect the synthesis of DNA. We induced the ISR and the consequent phosphorylation of eIF2alpha at Serine 51 by stimulating the kinases PERK, PKR, and GCN2, or by inhibiting GADD45A (regulatory subunit of the PP1 phosphatase) using the small compounds thapsigargin (Thap)^27^, BEPP-monohydrochloride^28^, l-Histidinol^29^, or Sephin^30^, respectively (Fig. 1a). Increased phosphorylation of eIF2alpha and elevated expression of ATF4 following treatment confirmed ISR activation in all cases (Fig. 1b; Supplementary Fig. S1A). Sephin inhibits the removal of constitutive phosphate modifications on eIF2alpha. This induces a moderate increase in phosphorylation of eIF2alpha, less pronounced than with Thap or BEPP, i.e., activators of eIF2alpha kinases. We first performed an EdU incorporation assay to measure overall DNA synthesis in individual cells upon ISR activation during S phase. As shown (Fig. 1c, d; Supplementary Fig. S1B), the activation of ISR using Thap or BEPP significantly reduced DNA synthesis in S phase. Then, we measured the progression of single DNA replication forks using DNA fiber assays, measuring the length of DNA tracks with incorporated IdU (Fig. 1e). Treatment with Thap led to a reduction in fork progression (Fig. 1f, g; Supplementary Fig. S1C, D). In addition, we found that treatment of U2OS cells with BEPP, Sephin, or l-Histidinol all impaired DNA fork progression significantly, albeit to different extents (Fig. 1h–l; Supplementary Fig. S1E–L). To understand whether the reduction in fork progression upon ISR was due to lower speed of DNA polymerase or a higher frequency of polymerase stalling, we conducted a 7-label fiber assay on Thap-treated cells (Fig. 1m) as described in our previous publications^9,10^. This revealed both increased stalling of DNA polymerase (i.e., decreased processivity) and slower DNA polymerization (Fig. 1n–p; Supplementary Fig. S1M).

Interestingly, despite the significant reduction in DNA replication progression following ISR stimulation, we did not observe a substantial increase in phosphorylation of Chk1 or histone variant H2AX (gamma H2AX) after 1 h (Supplementary Fig. S1N) or 4 h (Fig. 1q) as compared to gemcitabine, a well-established inducer of replicative stress^7^ indicating that the ISR slows down replication forks without triggering a strong DNA damage response. These results suggest that the ISR not only triggers a shutdown in protein synthesis but also imposes severe and immediate restrictions on DNA replication.

### Pharmacological antagonists of ISR partially rescue DNA replication

**Fig. 2:**
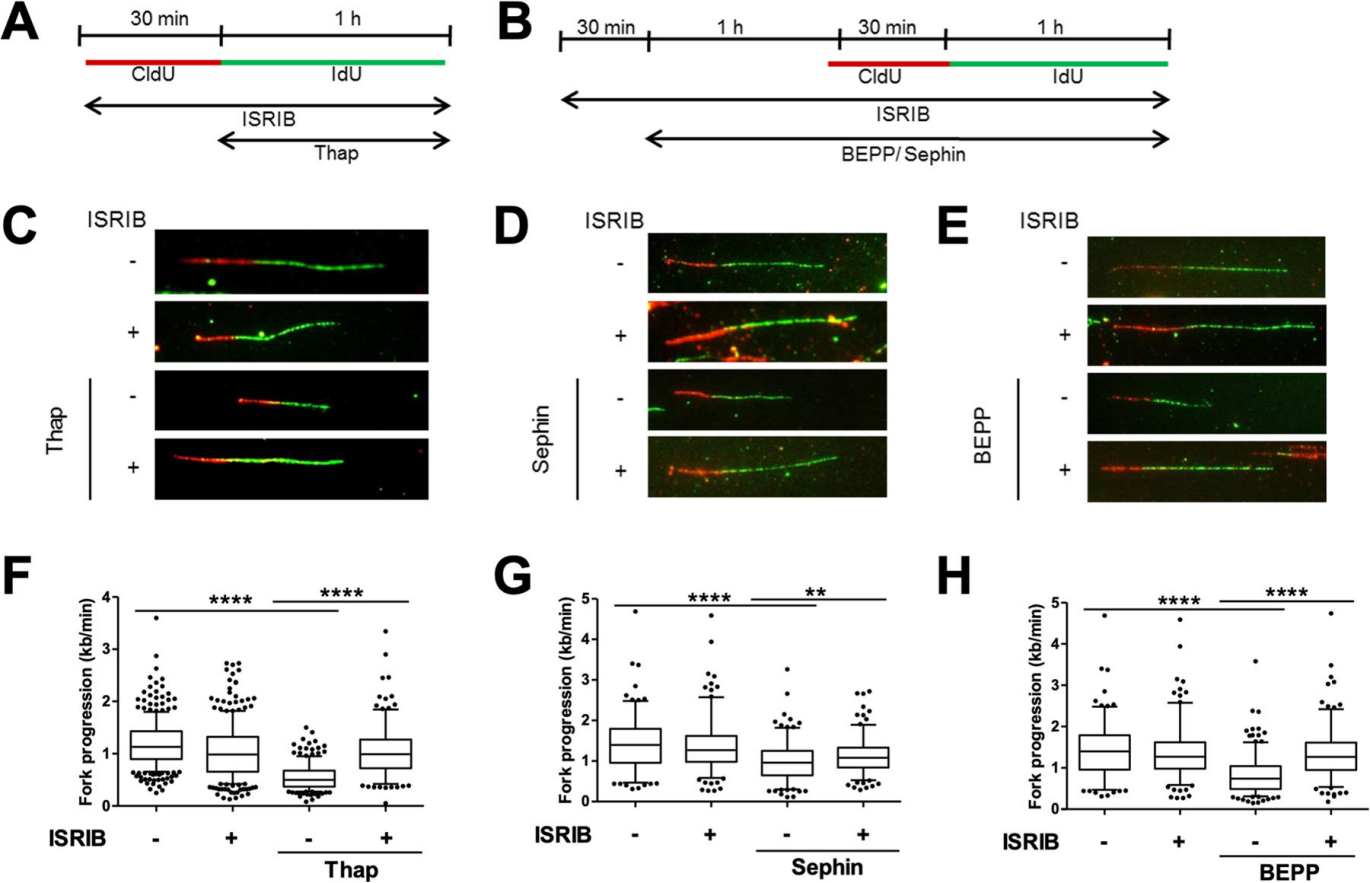
Pharmacological antagonists of ISR partially rescue DNA replication. **a** U2OS cells were treated with 1 μM ISRIB and at the same time incubated with CldU (30 min). Cells were labeled with IdU (60 min) in the presence of ISRIB and 4 μM Thap and then harvested for DNA fiber assay analysis. **b** Cells were pre-treated with 1 μM ISRIB for 30 min and then with 10 μM BEPP or 25 μM Sephin in the presence of ISRIB for 2.5 h. To label newly synthesized DNA, cells were incubated with CldU and IdU during the last 1.5 h as shown, and then harvested for analysis. **c**–**e** Representative DNA tracks as labeled in red (CldU) and green (IdU) of cells treated with ISRIB/Thap (**c**), ISRIB/Sephin (**d**), or ISRIB/BEPP (**e**). **f**–**h** Fork progression of IdU label of cells treated with ISRIB/Thap (**f**), ISRIB/Sephin (**g**) or ISRIB/BEPP (**h**) represented as 5–95 percentile box plots. Plots shown are a representative of two or three independent experiments. See Supplementary Fig. S2A–E.

Based on our findings suggesting that the ISR interferes with DNA replication, we now investigated whether these effects are downstream of phosphorylated eIF2alpha and could be reversed using a small molecule inhibitor of ISR known as ISRIB^31–33^ (Fig. 1a). ISRIB enhances the activity of the nucleotide exchange factor eIF2B, thereby overcoming the inhibitory effect of eIF2alpha phosphorylation. We pre-treated cells with ISRIB, followed by the ISR inducers Thap, BEPP or Sephin, and then measured DNA replication fork progression (Fig. 2a, b). Single treatment of cells with Thap, BEPP or Sephin resulted in an impairment of DNA replication as observed before, but pre-treatment of these cells with ISRIB significantly prevented this inhibition of DNA replication (Fig. 2c–h; Supplementary Fig. S2A–E). Similarly, inhibition of PERK with a pharmacological inhibitor, PERKi or GSK2606414^34^, was also able to significantly rescue DNA replication defects by Thap treatment (Supplementary Fig. S2F–J). Activation and inhibition of ISR were confirmed using ATF4 detection as readout (Supplementary Fig. S2K, L). These findings clarify that the compounds used interfere with DNA replication through the ISR and through eIF2alpha phosphorylation.

### Stimulation of the ISR induces R-loops

**Fig. 3:**
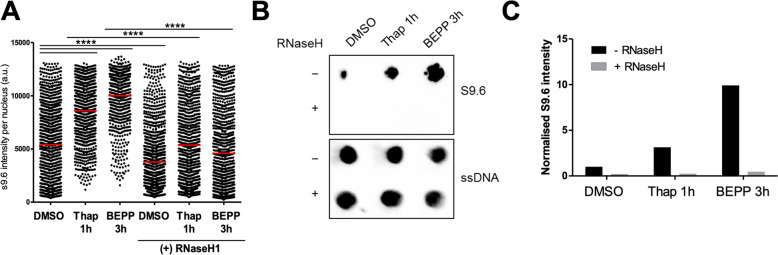
Stimulation of the ISR induces R-loops. **a** Cells were treated with 4 μM Thap or 10 μM BEPP for 1 or 3 h, respectively, with/without RNaseH1 overexpression prior to fixation and S9.6 immunofluorescence analysis. Scatter plot of S9.6 intensity per nucleus of cells (arbitrary units), determined by quantification from one of two independent experiments (Supplementary Fig. S3A, B). Red line represents mean nuclear S9.6 staining. **b** ISR was induced in cells using Thap (4 μM, 1 h) or BEPP (10 μM, 3 h), followed by dot blot analysis to quantify DNA:RNA hybrids. Equal amounts of DNA were spotted onto nitrocellulose membrane, and R-loops were detected using the S9.6 antibody. RNaseH treatment was conducted alongside and used as a negative control to confirm specificity of the signal. The signal of ssDNA was used as an internal sample loading control. See Supplementary Fig. S3D for a replicate. **c** The S9.6 signals obtained in **b** were quantified, normalized against the loading control (ssDNA signal), then against DMSO (without RNaseH) and plotted as bar charts. See Supplementary Fig. S3E.

We were now searching for a mechanism that allows the ISR to interfere with DNA replication. DNA:RNA hybrids (R-loops) have recently emerged as one of the major players in regulating DNA replication^15,17,35^. They are formed through the hybridization of newly synthesized RNA to its template DNA while looping out the opposite DNA strand. R-loops can pose as a steric hindrance to an oncoming replisome, thereby blocking DNA replication^16^. We investigated whether ISR induction led to an enrichment of R-loops. In cells treated with Thap or BEPP, we detected DNA:RNA hybrids by immunofluorescence with an antibody against them (S9.6) (Supplementary Fig. S3A)^19,20^. As a negative control, we overexpressed RNaseH1^20^ in these cells, i.e., an RNase that specifically removes the RNA portion of DNA:RNA hybrids (Supplementary Fig. S3A)^15^. By quantification, we found a significant increase in the intensity of S9.6 fluorescence in the nuclei of cells treated with Thap or BEPP (Fig. 3a; Supplementary Fig. S3B). Upon RNaseH1 overexpression, the S9.6 staining intensity within these nuclei decreased to intensities similar to control-treated cells (Fig. 3a; Supplementary Fig. S3B). We confirmed RNaseH1 overexpression and ISR induction by immunoblot analysis of RNaseH1 and ATF4 levels (Supplementary Fig. S3C). To supplement our immunofluorescence experiments, we performed dot blot analyses using the antibody S9.6. Cells were treated with Thap or BEPP, followed by chromatin preparation. Samples were also treated with RNaseH as a negative control. In each case, DNA:RNA hybrids were then detected on dot blots. Similar to the immunofluorescence, we observed a strong increase in S9.6 intensity upon ISR, which was abolished by RNaseH (Fig. 3b, c; Supplementary Fig. S3D, E). Thus, ISR activation leads to an enrichment of R-loops.

### Removal of R-loops re-establishes DNA replication upon induction of ISR but compromises survival of stressed cells

**Fig. 4:**
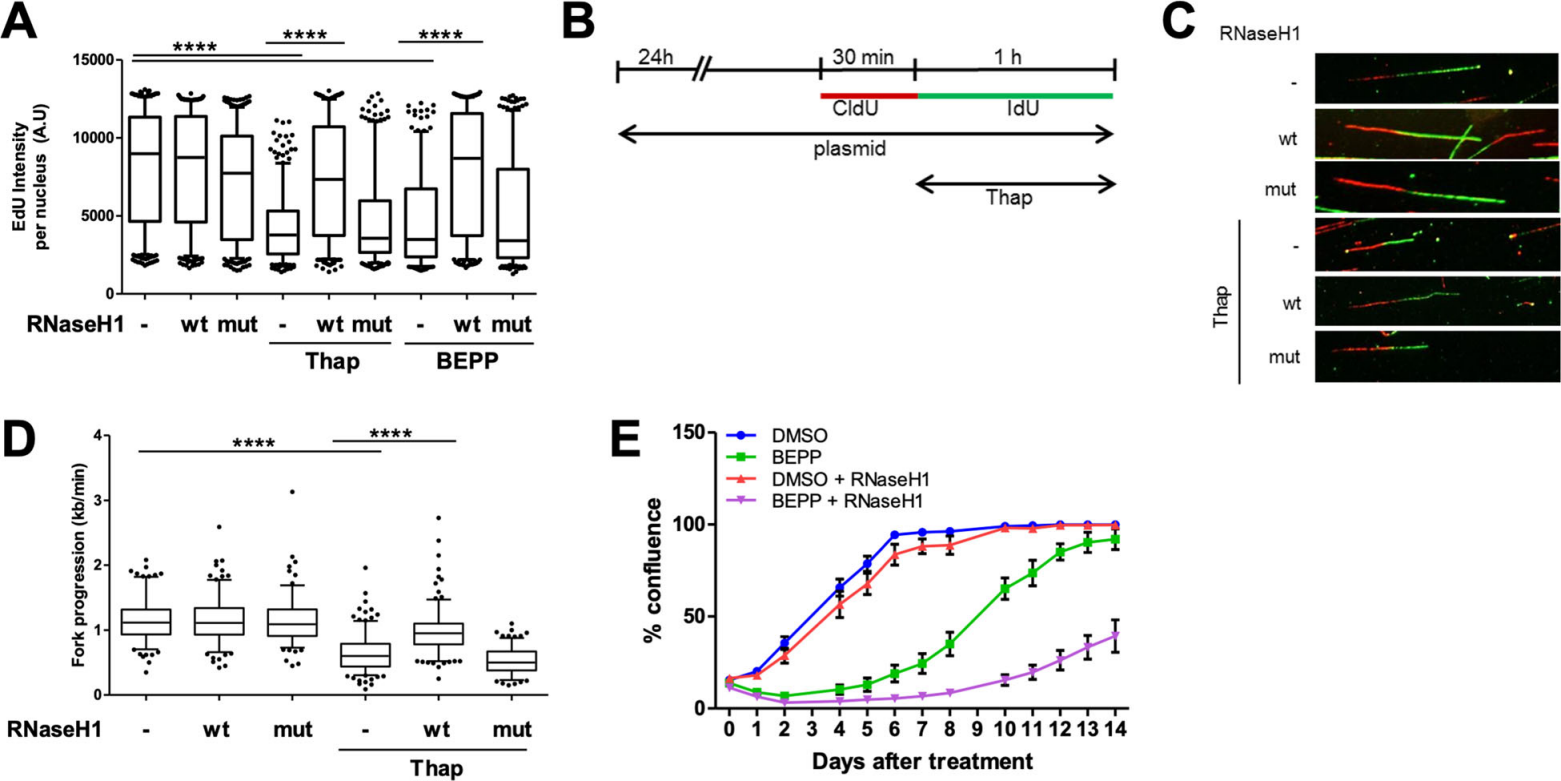
Removal of R-loops re-establishes DNA replication upon induction of ISR but compromises survival of stressed cells. **a** U2OS cells transfected with control, RNaseH1 wildtype (wt), or catalytically mutant RNaseH1 (D10R-E48R) (mut) expression plasmids for 24 h were treated with Thap (4 μM, 1 h) or BEPP (10 μM, 2.5 h). During the last 1 h of treatment, the cells were labeled with 20 μM EdU and then subjected to fluorescence analysis of EdU incorporation. Box plot (5–95 percentile whiskers) of the quantified EdU intensities per nucleus of one of three independent experiments. For replicates, see Supplementary Fig. S4 B, C. **b** Transfection of cells with control or RNaseH1 plasmids (wt or mut) were conducted as described in a 24 h prior to labeling with CldU (30 min) and IdU (60 min). Cells were treated with 4 μM Thap during the IdU label and then harvested for analysis. **c** Representative DNA fiber tracks stained for CldU (red) and IdU (green) of Thap–treated cells overexpressing the respective plasmids as described in b. **d** Box plot (5–95 percentile whiskers) of DNA fork progression of cells overexpressing RNaseH1 wt or mut plasmids in the presence/absence of Thap. Fork progression was measured using the IdU label and the plot shown is a representative of one out of three independent experiments. See Supplementary Fig. S4 D, E. **e** Long-term proliferation assay of BEPP-treated cells with/without RNaseH1 overexpression displayed as percentage confluence. Transfected cells that were synchronized at S phase were treated with either DMSO or BEPP (30 μM) for 6 h. The media was then replenished and cell confluency at day 0 was measured using the Celigo Cytometer. Confluency was measured on the indicated days for 2 weeks. Mean ± SD of technical duplicates were plotted. Plot is a representation of three biological repeats (Supplementary Fig. S4M, N).

As ISR activation induced more R-loops, we hypothesized that these R-loops were responsible for compromising DNA replication. To test this, we first overexpressed wildtype or catalytically mutant RNaseH1^19^ and treated these cells with Thap or BEPP to induce ISR, and measured total DNA synthesis by EdU incorporation. As seen previously (Fig. 1c, d), EdU incorporation was reduced upon ISR (Fig. 4a; Supplementary Fig. S4A–C). Strikingly, we now observed that the overexpression of catalytically active RNaseH1 largely restored EdU incorporation and thus DNA synthesis in both Thap and BEPP-treated cells (Fig. 4a; Supplementary Fig. S4A–C). To test whether removal of R-loops was also able to rescue single DNA fork progression, we subjected cells overexpressing wildtype or catalytically inactive RNaseH1 and treated with Thap or BEPP to DNA fiber assay analysis (Fig. 4b; Supplementary Fig. S4F). The removal of R-loops with wildtype but not mutant RNaseH1 completely rescued DNA replication in the context of ISR (Fig. 4c, d; Supplementary Fig. S4D–J). Immunoblot analysis confirmed that RNaseH1 overexpression did not interfere with eIF2alpha phosphorylation (Supplementary Fig. S4K, L) and thus not with the ISR per se. We then hypothesized that R-loop induction and the resulting impairment of DNA replication upon ISR might help cells to survive by halting the complex DNA replication program in the face of stress conditions. To investigate whether the inhibition of DNA replication following accumulation of R-loops upon ISR is protective to the cell, we conducted a proliferation assay of cells treated with BEPP in the presence or absence of RNaseH1. Indeed, removal of R-loops via the overexpression of RNaseH1 further reduced proliferation of cells compared to cells that were treated with BEPP alone (Fig. 4e; Supplementary Fig. S4M, N). Our findings therefore suggest that ISR impairs DNA replication through inducing R-loops and that this inhibition in DNA replication is supporting cell survival during stress in U2OS cells.

### Ongoing transcription is required for compromising DNA replication by the ISR

**Fig. 5:**
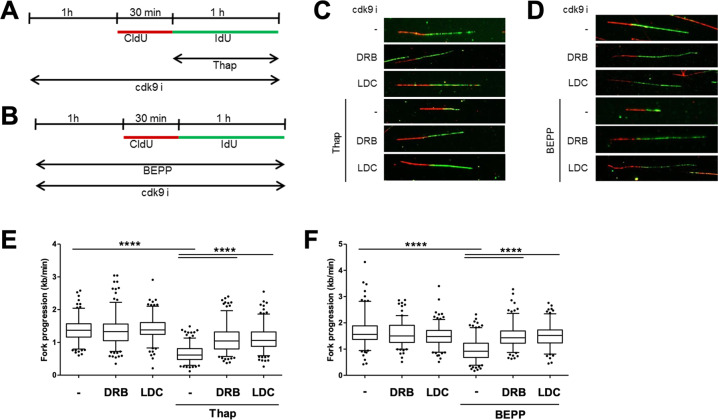
Ongoing transcription is required for compromising DNA replication by the ISR. **a** Cells were pre-treated with CDK9 inhibitors (25 μM DRB, 10 μM LDC067) for 1 h prior to labeling with CldU (30 min) and IdU (60 min) in the presence of CDK9i and Thap (4 μM). Cells were then harvested for DNA fiber analysis. **b** U2OS cells were treated with CDK9i (25 μM DRB, 10 μM LDC067) and BEPP (10 μM) for 1 h and then labeled with CldU (30 min) and IdU (60 min) with both CDK9i and BEPP prior to analysis. **c**, **d** Representative DNA fiber tracks of cells treated with CDK9i/Thap (**c**) or CDK9i/BEPP (**d**) visualized via immunostaining of CldU (red) and IdU (green). **e**, **f** IdU tracks of cells treated with CDK9i and Thap (**e**) or CDK9i and BEPP (**f**) were used to measure fork progression and presented as box plots (5–95 percentile whiskers). One representative plot from three independent experiments shown. See Supplementary Fig. S5A–D.

R-loops were suggested to form between RNA and its DNA template, shortly after transcription^15^. This raised the hypothesis that short-term inhibition of transcription should prevent R-loop accumulation and hence avoid replication impairment in the context of the ISR. To test this, we employed two different CDK9 inhibitors, DRB^36^ and LDC067^37^. CDK9 inhibition is an established way to interfere with the elongation of transcription^38^. We measured DNA replication of cells treated with Thap or BEPP, in the presence or absence of CDK9 inhibitors (Fig. 5a, b). And indeed, the inhibition of transcription significantly rescued DNA replication from its impairment by ISR (Fig. 5c–f; Supplementary Fig. S5A–D), suggesting that ongoing transcription and R-loops formed by ISR are responsible for impairing DNA replication.

### ISR activation blocks the synthesis of histones required for DNA replication

**Fig. 6:**
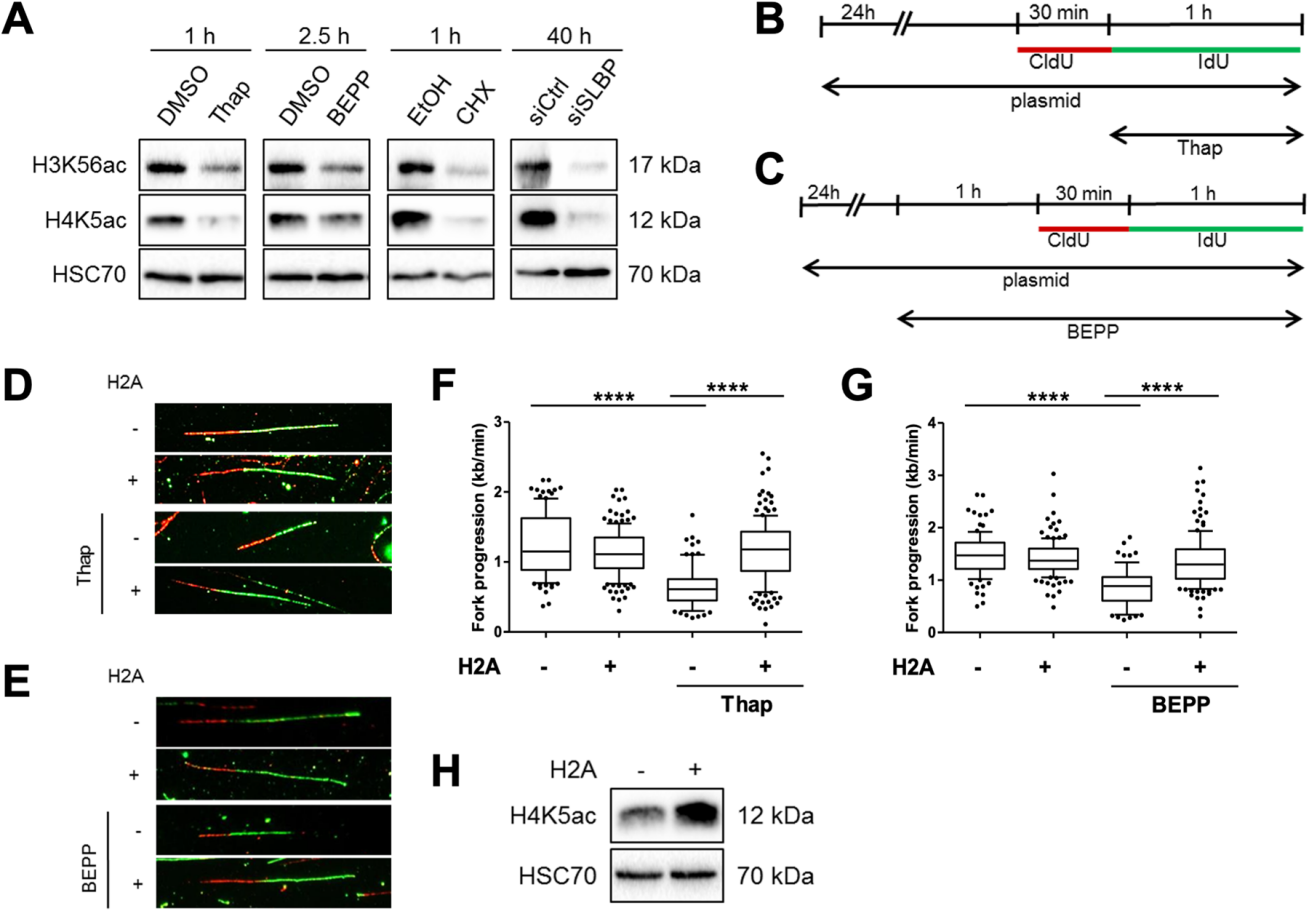
ISR activation blocks the synthesis of histones required for DNA replication. **a** Soluble proteins were extracted from cells treated with Thap (4 µM), BEPP (10 µM), CHX (50 µg/ml) or cells transfected with siRNA against SLBP (100 nM). Immunoblot analyses of soluble histone-3 lysine-56 acetylation (H3K56ac) and histone-4 lysine-5 acetylation (H4K5ac) were used to measure newly synthesized histones^12,41^. HSC70 as loading control. **b** Cells were transfected with H2A or control plasmids, labeled with CldU (30 min) followed by IdU (60 min). Cells were treated with Thap (4 μM) during the IdU label as indicated prior to analysis. **c** Cells were transfected with H2A or empty vector plasmids and treated with BEPP for 2.5 h. Newly synthesized DNA was labeled with CldU (25 μM, 30 min) followed by IdU (250 μM, 60 min) during the last 1.5 h in the presence of BEPP then harvested for analysis. **d** Representative DNA fiber tracks of cells transfected with plasmids (control, H2A) and labeled as described in **b**. **e** Images of DNA fibers (representative) of BEPP-treated cells overexpressing control or H2A plasmids visualized as CldU (red) and IdU (green). **f** Fork progression (kb/min) of cells in **d** calculated using IdU track length. DNA fork progression displayed as box plot (5–95 percentile whiskers) and is a representative data of one of three independent experiments. See Supplementary Fig. S6J, K. **g** DNA fork progression (kb/min) of cells treated as in **c** and displayed as box plots (5–95 percentile whiskers). IdU label was used to calculate fork progression. Data are representative of three independent experiments. See Supplementary Fig. S6L, M. **h** Western blot analysis of soluble H4K5ac from H2A-overexpressing cells. HSC70 used as loading control.

Phosphorylation of eIF2alpha at Ser51 during ISR inhibits cap-dependent translation, thereby blocking the synthesis of most proteins in the cell. To investigate whether abolished protein synthesis is sufficient to impair DNA replication, we treated cells with a well-established ribosome inhibitor, cycloheximide (CHX), and measured DNA replication progression (Supplementary Fig. S6A). Within an hour of CHX treatment, we observed a strong reduction in DNA replication progression (Supplementary Fig. S6B–D), mimicking the effects we observed with the ISR inducers (Fig. 1e–l; Supplementary Fig. S1C–L). Next, we asked which kind of proteins need to be synthesized continuously to sustain DNA replication. Based on previous reports^11,12,23,39^, we suspected that histones need to be provided throughout DNA synthesis to avoid replication stress. Indeed, inducing the ISR by Thap or BEPP quickly reduced the levels of newly synthesized soluble histones, as marked by acetylation of lysine residue 56 on Histone-3 (H3K56ac) or lysine residues 5 or 12 on Histone-4 (H4K5ac or H4K12ac)^12,40–43^, to a similar extent as upon CHX treatment (Fig. 6a; Supplementary Fig. S6E). To test whether a reduction in histone synthesis alone is sufficient to hinder DNA replication in our system as found earlier^12^, we used siRNA to deplete the stem loop-binding protein (SLBP) that is required for translation of histones. As expected, SLBP depletion also resulted in a mark decrease in soluble H3K56ac, H4K5ac, and H4K12ac (Fig. 6a; Supplementary Fig. S6E). Of note, a significant impairment in DNA replication was observed by SLBP depletion alone (Supplementary Fig. S6F–I), strongly suggesting that histones are the critical protein species the reduced synthesis of which is responsible for impaired DNA replication during ISR.

To find out whether restoring histone levels alone might allow DNA replication even during ISR, we measured DNA replication in cells overexpressing histone H2A and treated with Thap or BEPP (Fig. 6b, c). Strikingly, overexpression of histone H2A restored DNA replication despite ISR activation (Fig. 6d–g; Supplementary Fig. S6J–O). As shown in Fig. 6a, the ISR led to a general decrease in newly synthesized histones, making it difficult at first glance to explain how the overexpression of H2A alone could rescue DNA replication upon ISR. We hypothesized that the overexpression of one histone (H2A in this case) could increase the levels of other free histones, e.g., by forming stable histone complexes. Indeed, we also observed an increase in histone H4 carrying an acetylation of lysine-5 upon H2A overexpression (Fig. 6h). This modification is typically found on newly synthesized H4^12^. Taken together, we conclude that the ISR interferes with DNA replication at least in part through inhibiting histone synthesis.

### Inhibition of histone synthesis induces R-loops, which impairs DNA replication

**Fig. 7:**
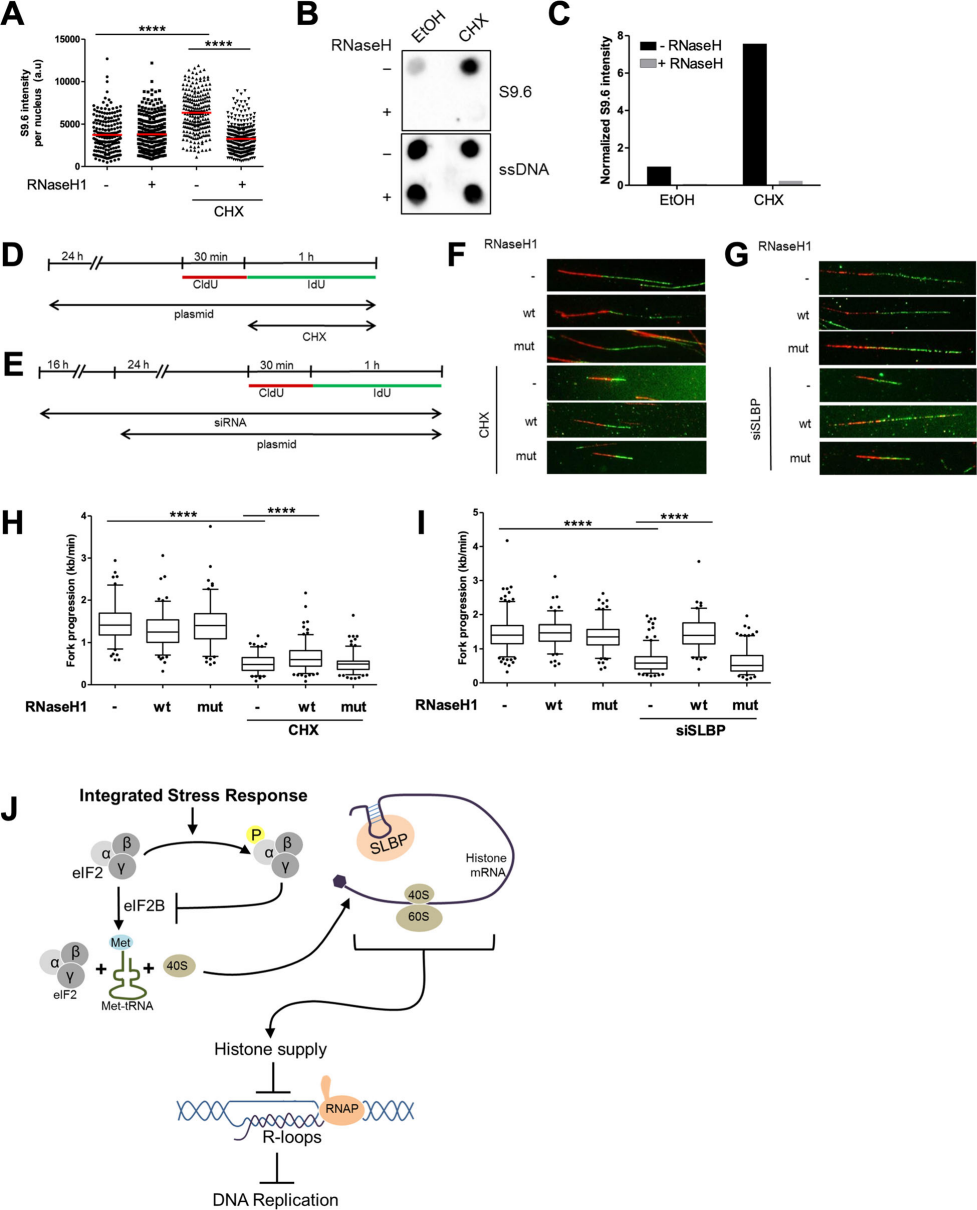
Inhibition of histone synthesis induces R-loops, which impairs DNA replication. **a** Cells synchronized at S phase and transfected with either control or RNaseH1 expression plasmids were treated with CHX (50 µg/ml) for 1 h, then harvested for S9.6 immunofluorescence analysis as described in Fig. 3a. Intensity of S9.6 staining per nucleus was quantified and displayed as a scatter plot. Red line represents mean S9.6 intensity per nucleus. See Supplementary Fig. S7B for a replicate. **b** Dot blot analysis of S phase cells to detect R-loops using S9.6 antibody. Synchronized cells in S phase were treated with CHX (50 μg/ml, 1 h) and harvested. Equal amounts of DNA were spotted onto nitrocellulose membrane. R-loops were detected using S9.6 antibody whereas subsequent ssDNA detection (on denatured DNA) was used as an internal loading control. As a negative control, samples were treated with RNaseH enzyme for 3 h at 37 °C. Blot is a representative of two independent experiments. See Supplementary Fig. S7D. **c** Signal from the spots in **b** were quantified and normalized to the loading control (ssDNA) and then to the sample without ISR or RNaseH treatment. See Supplementary Fig. S7E. **d** U2OS cells were transfected with plasmids (control, RNaseH1 wt or RNaseH1 mut) 24 h prior to labeling with CldU (30 min) and IdU (60 min). CHX (50 μg/ml) was added to the cell during the IdU label. **e** Cells were transfected with siRNA (siCtrl or siSLBP, 100 nM) 16 h prior to overexpression with plasmids (control, RNaseH1 wt, or RNaseH1 mut). Cells were then incubated with CldU (30 min) and IdU (60 min) to label newly synthesized DNA and then harvested. **f**, **g** DNA fiber tracks of CHX-treated cells (**f**) or cells depleted from SLBP (**g**) with overexpression of either the control, RNaseH1 wt, or RNaseH1-mutant plasmids. Fiber tracks were observed by immunostaining of CldU (red) and IdU (green). **h**, **i** Box plot (5–95 percentile whiskers) showing the fork progression as measured using IdU track length of CHX-treated (**h**) or SLBP–depleted (**i**) cells in the presence/absence of RNaseH1 overexpression. Representative data shown from one of three independent experiments. See Supplementary Fig. S7F–I. **j** Graphical abstract: The integrated stress response inhibits DNA replication through blocking histone synthesis and inducing R-loops. Supplementing the cells with histones rescues DNA replication impairment upon the ISR. In addition, removal of R-loops upon the ISR or histone depletion both restores DNA replication fork progression.

We have found that the ISR blocks histone synthesis, which compromises DNA replication (Fig. 6). Moreover, the ISR can induce R-loops (Fig. 3), which are also required to perturb DNA replication (Figs. 4 and 5). Therefore, we hypothesized that histone deprivation induces the formation of R-loops, which then compromises DNA replication. To investigate this, we performed immunofluorescence staining using the S9.6 antibody to detect R-loops on S phase cells treated with CHX. CHX, can be expected to block the synthesis of histones (and other proteins). These analyses were carried out with and without RNaseH1 overexpression. Indeed, CHX-treated cells accumulated DNA:RNA hybrids (Fig. 7a; Supplementary Fig. S7A–C). Similarly, dot blot analysis using the S9.6 antibody on chromatin from these cells also revealed a profound induction of R-loops, which was removed upon RNaseH treatment (Fig. 7b, c; Supplementary Fig. S7D, E). Next, to investigate whether DNA replication impairment by histone depletion could also be restored by removing R-loops, we depleted cells of new histones using CHX or by siRNA to SLBP, in the presence or absence of either wildtype or catalytically inactive RNaseH1, and then measured the progression of DNA replication (Fig. 7d, e). We observed that overexpression of wildtype RNaseH1 but not its mutant rescued DNA replication upon histone depletion, albeit to different extents in the CHX-treated cells vs cells depleted of SLBP (Fig. 7f–i; Supplementary Fig. S7F–K). Together, these results suggest a mechanistic concept of ISR-induced DNA replication impairment. Accordingly, ISR blocks histone synthesis, which then interferes with DNA replication, at least in part through the accumulation of R-loops.

## Discussion

Our results indicate that the ISR compromises DNA replication, within the first hour of eIF2alpha phosphorylation, and through the depletion of histones, in U2OS cells. When new histones become unavailable, by ISR or histone chaperone inhibition, R-loops generated mediate the impairment of DNA replication fork progression (Fig. 7j).

Is this replication stress? Previous reports suggest that the depletion of histones slow down replication fork progression, but do not detectably trigger the activation of Chk1, a classical hallmark of replication stress^6,12,23^. Similarly, in our hands, Chk1 phosphorylation or phosphorylation of the histone variant H2AX (gamma H2AX) are observed only to a low extent (when compared to treatment with the nucleoside analog gemcitabine) (Fig. 1q; Supplementary Fig. S1N). Notably, the Chk1 phosphorylation status upon the ISR was compared directly to a standard replicative stress inducer. In line with this, it is possible that ISR induction could activate Chk1 as seen in a previous study^22^, but only moderately. Taken together with the observed accumulation of R-loops, we propose that R-loops generated upon the ISR as such are not sufficient to strongly activate Chk1, despite interfering with the progression of DNA replication forks, at least not within the first 4 h of interfering with DNA replication^12^.

It was previously reported that the lack of histone supply hinders replication fork progression^11,12,23^. The mechanism(s) were suggested to include interactions of histones with the MCM helicase and/or the delayed removal of PCNA from Okazaki fragments but remain to be fully clarified^12^. On the other hand, one study in a different cell system has shown enhanced DNA replication upon histone depletion^44^, suggesting that not all cell types may respond uniformly to histone depletion. Importantly however, our results are in agreement with those shown in previous works, also using U2OS cells^11,12,23^, and we additionally provide the following possible mechanism. We hypothesize that when histones are missing, nucleosome-free DNA accumulates. This provides more opportunities for DNA:RNA hybridization (Fig. 7a–c). In line with that, we propose that the resulting R-loops are one of the causes for the observed replication fork impairment in our system, since RNaseH1 enhanced DNA synthesis in the context of histone depletion (Fig. 7d–i). Curiously, although significant, the restoration of DNA replication upon RNaseH1 overexpression in CHX-treated cells was less impressive compared to cells treated with ISR inducers (Fig. 7f, h). CHX is a broader and more complete translation inhibitor compared to the ISR, which only inhibits translation of a proportion of mRNAs^1^. Hence, it is possible that CHX may block the expression of many proteins not specific to R-loop homeostasis. Thus, to a greater extent than ISR induction or SLBP depletion, CHX may cause replicative defects, which are not solely due to R-loop accumulation.

It is important to note that the inhibition of translation by ISR could also promote R-loop accumulation due to the downregulation of proteins other than histones, which might as well be involved in maintaining R-loop homeostasis. Moreover, DNA replication stress could also lead to R-loop accumulation, perhaps leading to mutual enhancement^20^. Nevertheless, it remains to be determined how exactly the R-loops that form upon the ISR lead to stalled DNA replication. Apart from physical collisions, the accumulation of R-loops might trigger signaling pathways that attenuate fork progression^18^. Indeed, it has been shown that R-loops induce the phosphorylation of histone H3 at Ser10 (H3S10), a mark of chromatin compaction^45^. It is thus possible that the R-loops formed could lead to torsional stress throughout the DNA surrounding them through chromatin condensation, which then signals the replication machinery ahead to stop replicating DNA^16^.

In terms of physiological relevance, we propose that the inhibition of DNA replication as part of the ISR provides an advantage for cell survival, at least in our system. Under conditions of nutrient deprivation, it is conceivably advantageous that protein synthesis is reduced to a minimum. On top of this, our results in U2OS cells show that slowing down DNA synthesis through R-loop accumulation, as a newly established part of the ISR, helps the cell to survive nutrient restriction. This can be seen with a substantial impairment in proliferation of cells overexpressing RNaseH1 under ISR stimulation (Fig. 4e). After all, replicating a diploid human genome within one cell requires 2 × 3 × 10^9^ deoxynucleoside-triphosphates, each of which contains two energy-rich anhydride bonds. Stalling replication forks reduces the rate by that dNTPs are used and might thus contribute to survival under conditions of limited available energy. This might have contributed to the evolution of a tight coupling mechanism that immediately shuts down DNA synthesis in the context of ISR.

The ISR has also been suggested as a target for cancer therapy^46,47^. The idea is mainly to exacerbate proteotoxicity and the accumulation of unfolded proteins in cancer cells by inhibitors of kinases that would otherwise stimulate the ISR. Based on the results presented here, it is possible that interfering with the ISR may also overcome the stalling in DNA replication, perhaps enhancing the vulnerability of cancer cells toward drugs that provoke replication stress, e.g., nucleoside analogs or ATR inhibitors^6^. This suggests the use of ISR inhibitors with nucleoside analogs and/or ATR inhibitors in an attempt to achieve synergistic responses to eliminate cancer cells.

Proteasome inhibitors and HSP90 inhibitors form part of a general strategy to eliminate cancer cells by targeting essential cellular machineries^48^, or exploiting non-oncogene addiction^49–51^. However, these inhibitors can induce the ISR as well^52^. The results presented here suggest that this will also halt DNA replication forks. It remains to be determined whether this will diminish the activity of DNA-damaging chemotherapeutics toward cancer cells. In such a case, the simultaneous administration of proteotoxic drugs with certain conventional chemotherapeutics might need to be avoided to prevent drug antagonisms. On the other hand, the addition of an ISR inhibitor might restore the cooperation of a proteotoxic and a DNA-damaging drug.

In contrast to the direction explored here, replication stress can also induce the ISR, as has been reported in the case of the nucleoside analog gemcitabine^53^. Of note, however, gemcitabine was found to induce eIF2alpha phosphorylation with a delay of at least 6 h. In accordance with this, we were also unable to detect eIF2alpha phosphorylation within shorter periods of time upon gemcitabine treatment. Thus, the ISR probably does not affect the immediate response of cells toward direct triggers of replication stress. However, upon long-term application of chemotherapy, the ISR might represent a mechanism of cell resistance, not only by avoiding proteotoxic stress but also by slowing down DNA replication.

Another important aspect of the ISR consists in the defense against virus infection, in particular through activation of the kinase PKR^54,55^. Most obviously, this will reduce the production of virus proteins, e.g., for building new virus particles. Our results suggest that, in addition, DNA synthesis is diminished. On top of cellular DNA, this may also pertain to viral genomes, especially when they are associated with nucleosomes and thus require histone synthesis. This packaging of viral DNA into nucleosomes has been observed^56–58^. It is therefore tempting to speculate that the ISR might also contribute to a decrease in the synthesis of viral DNA, perhaps antagonizing virus production more efficiently than through translational shutdown alone.

## Supplementary information

Supplementary figure 1

Supplementary figure 2

Supplementary figure 3

Supplementary figure 4

Supplementary figure 5

Supplementary figure 6

Supplementary figure 7

Supplementary figure legends

Dataset 1: Supplementary table 1 - Fiber assay summary

Dataset 2: Supplementary table 2: fiber assay raw data

Supplementary table legends
