# Supplementary figures and images for: The integrated stress response induces R-loops and hinders replication fork progression
Source: Cell Death Dis. 2020 Jul 16;11(7):538. doi: 10.1038/s41419-020-2727-2 (PMC7366693; doi:10.1038/s41419-020-2727-2)

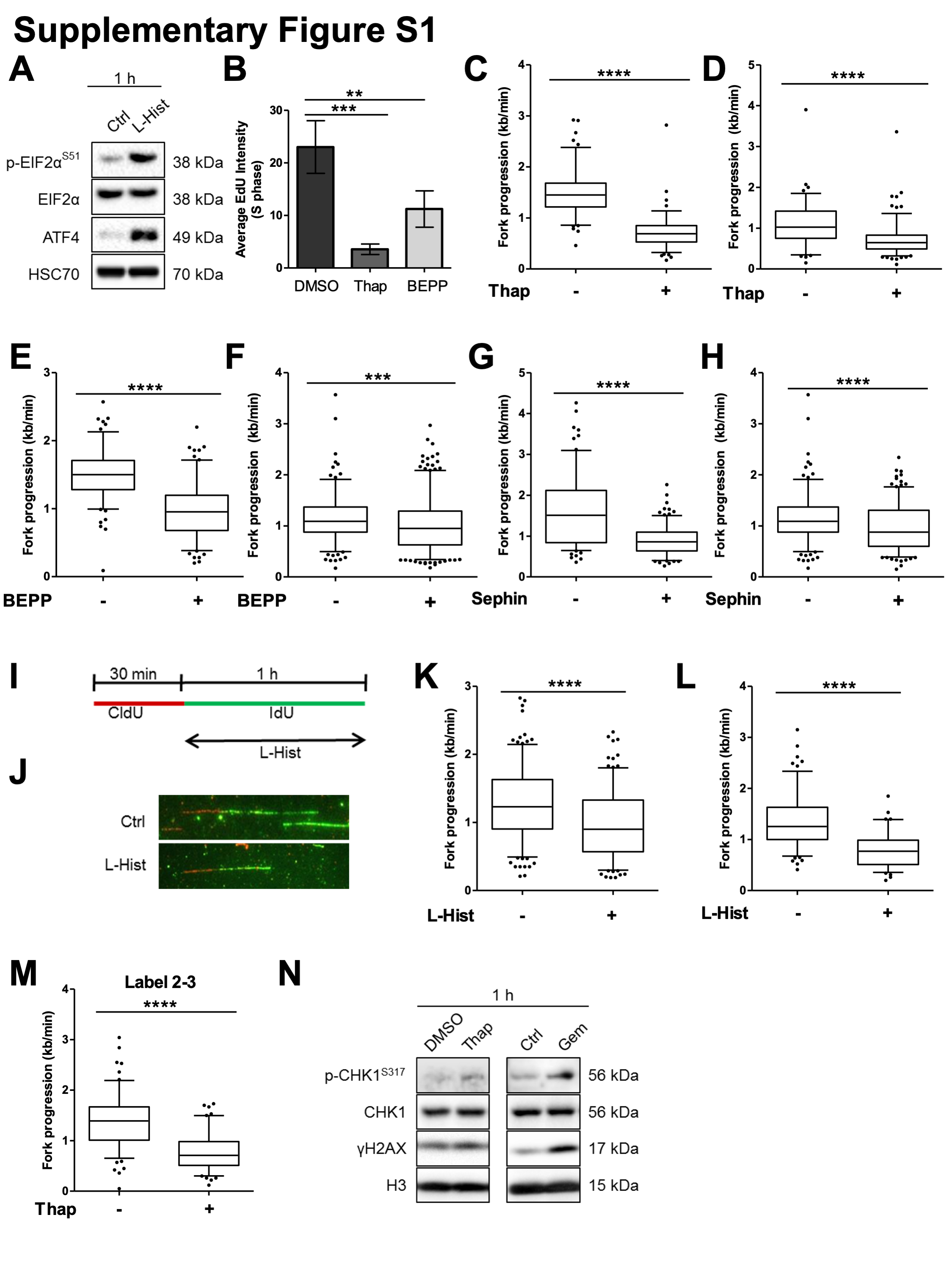

Supplement: Supplementary file 1 — Supplementary figure 1 [file 41419_2020_2727_MOESM1_ESM.tif]

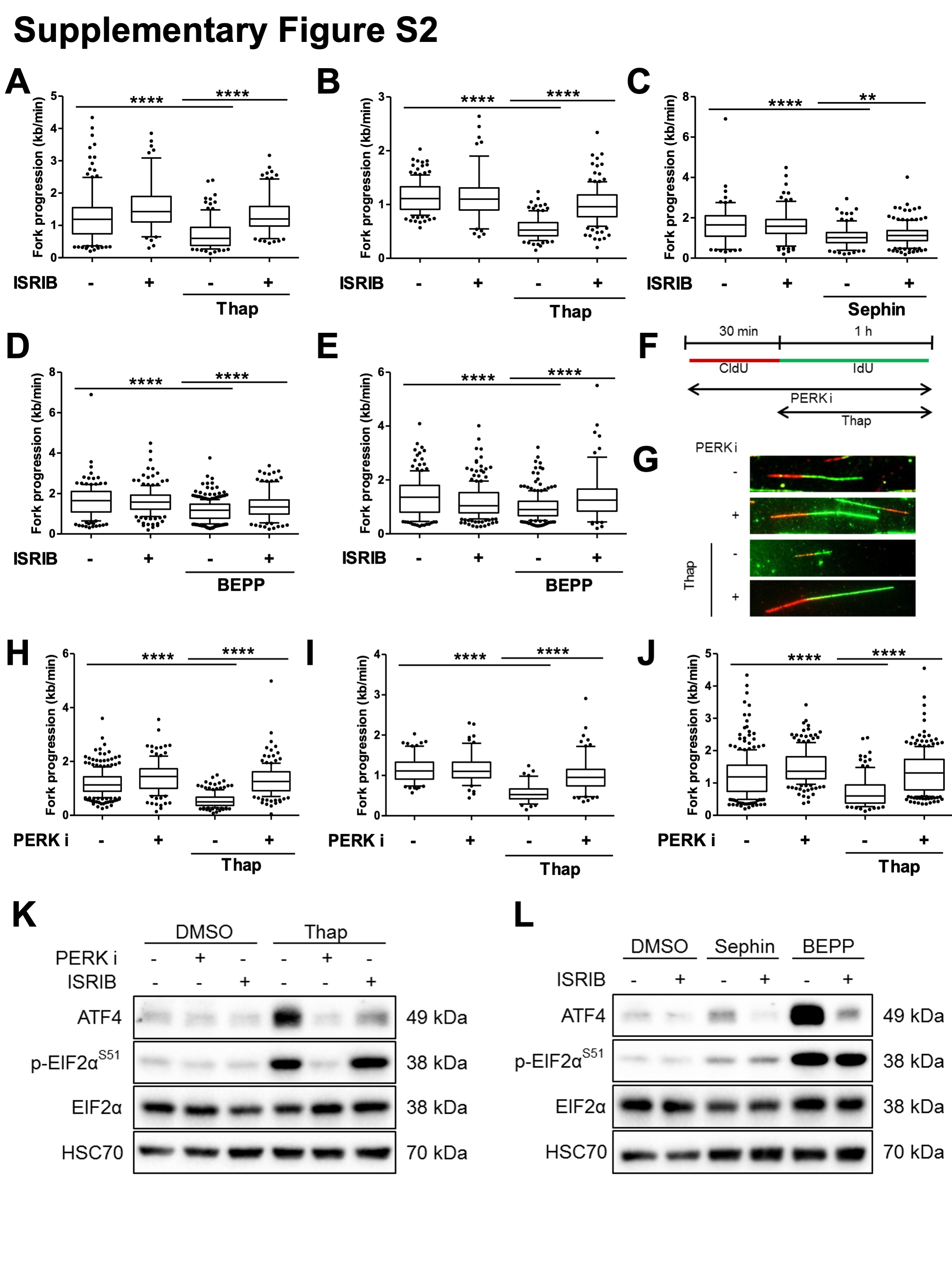

Supplement: Supplementary file 2 — Supplementary figure 2 [file 41419_2020_2727_MOESM2_ESM.tif]

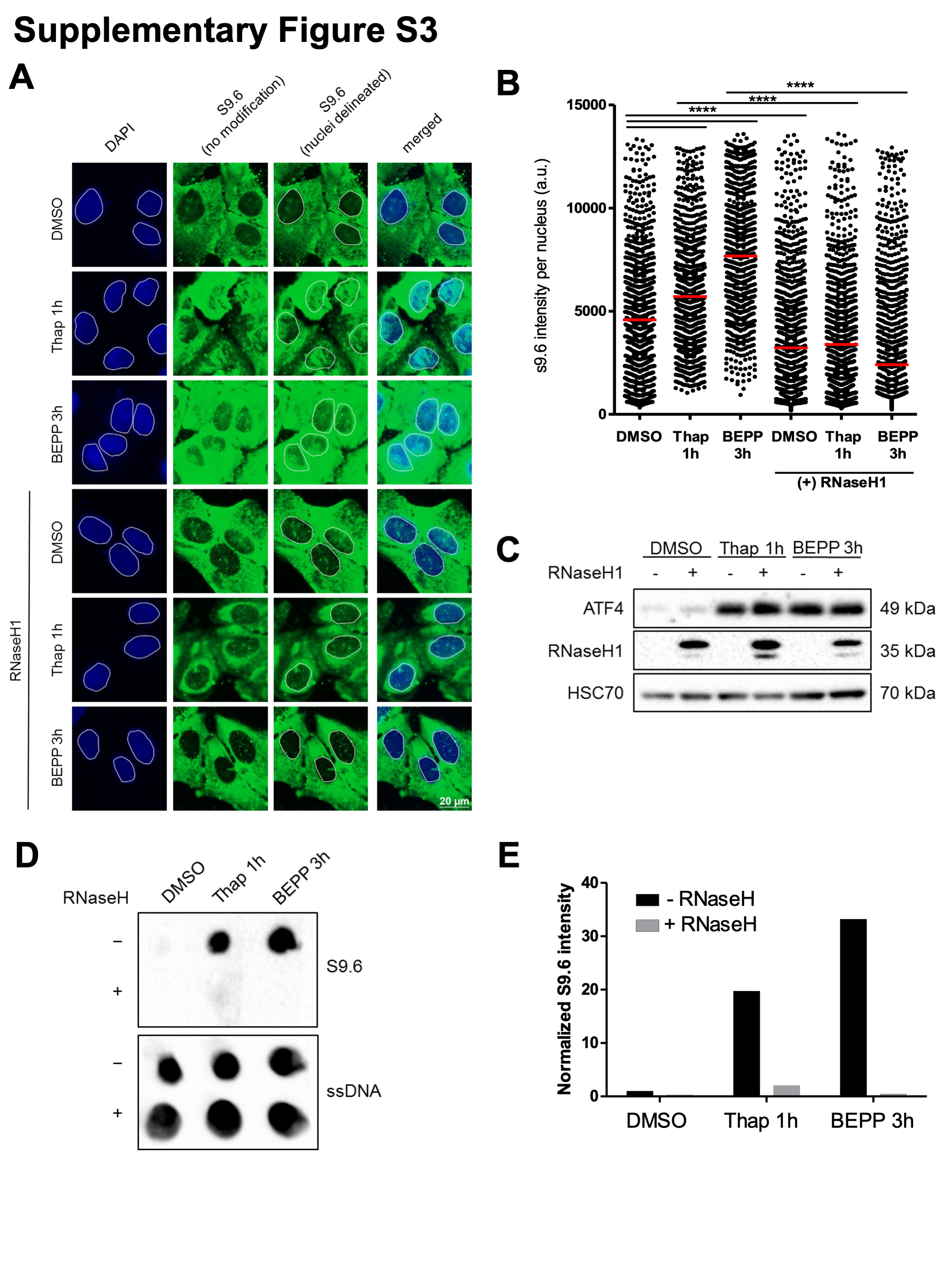

Supplement: Supplementary file 3 — Supplementary figure 3 [file 41419_2020_2727_MOESM3_ESM.tif]

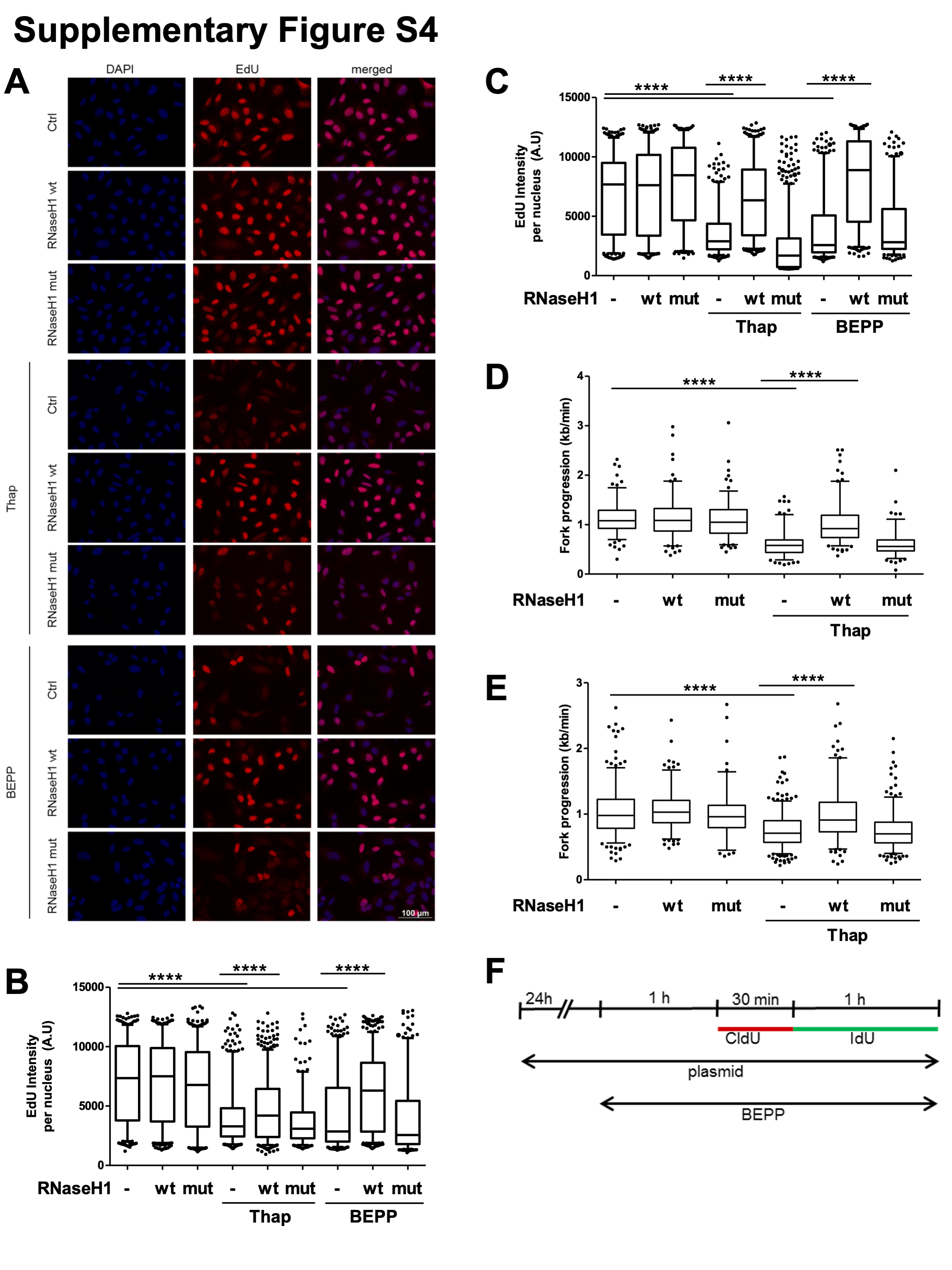

Supplement: Supplementary file 4 — Supplementary figure 4 [file 41419_2020_2727_MOESM4_ESM.tif]

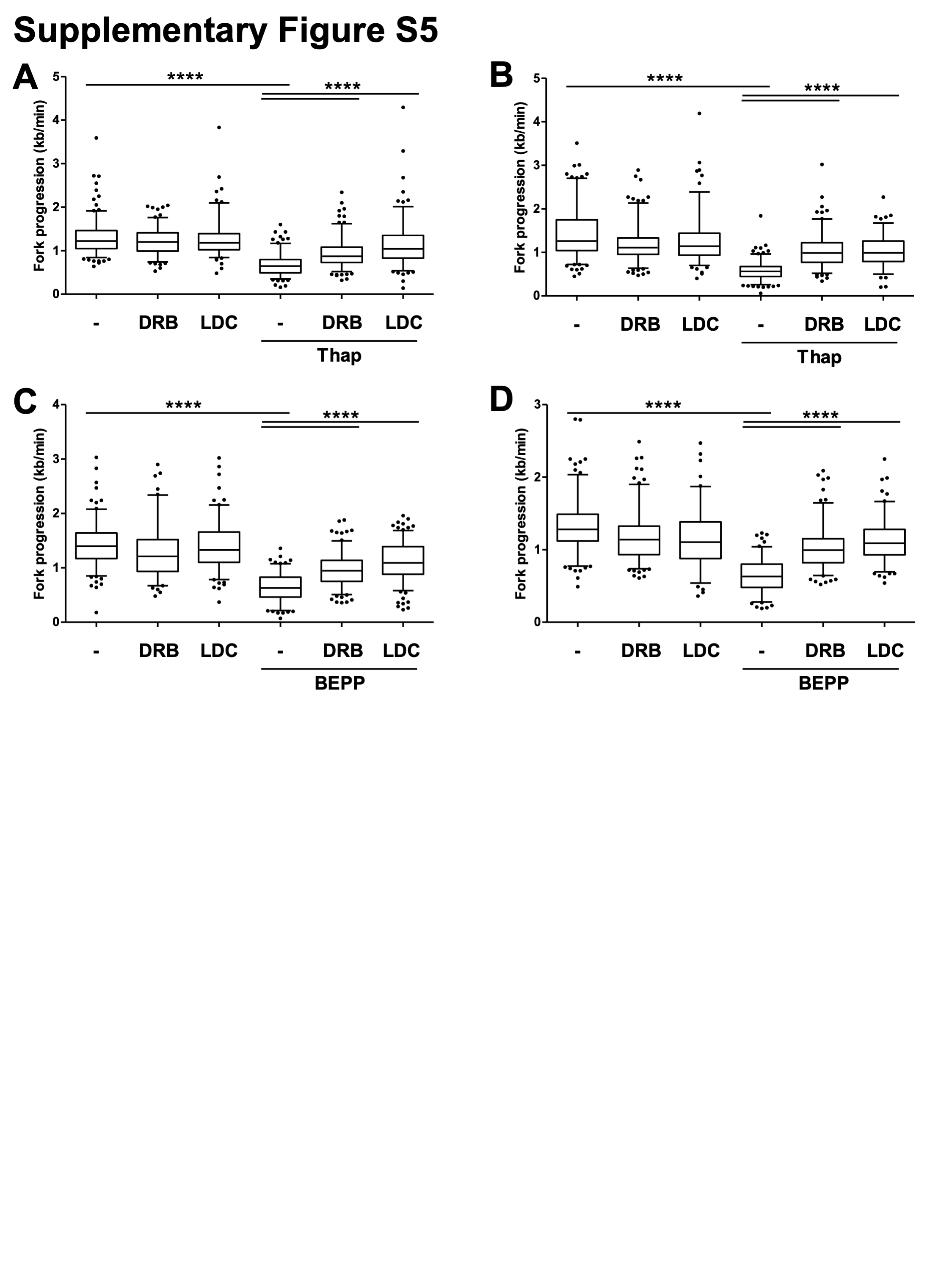

Supplement: Supplementary file 5 — Supplementary figure 5 [file 41419_2020_2727_MOESM5_ESM.tif]

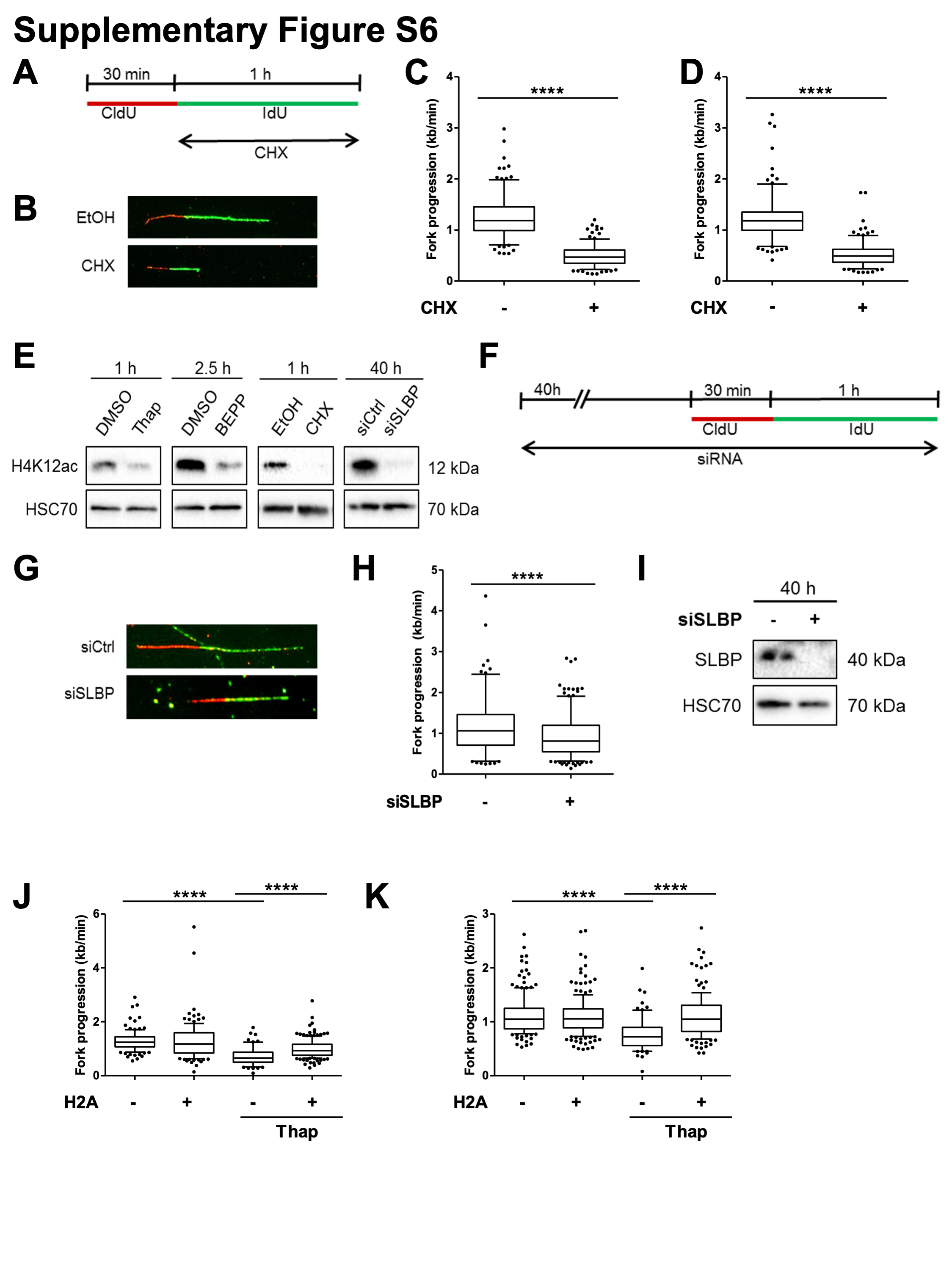

Supplement: Supplementary file 6 — Supplementary figure 6 [file 41419_2020_2727_MOESM6_ESM.tif]

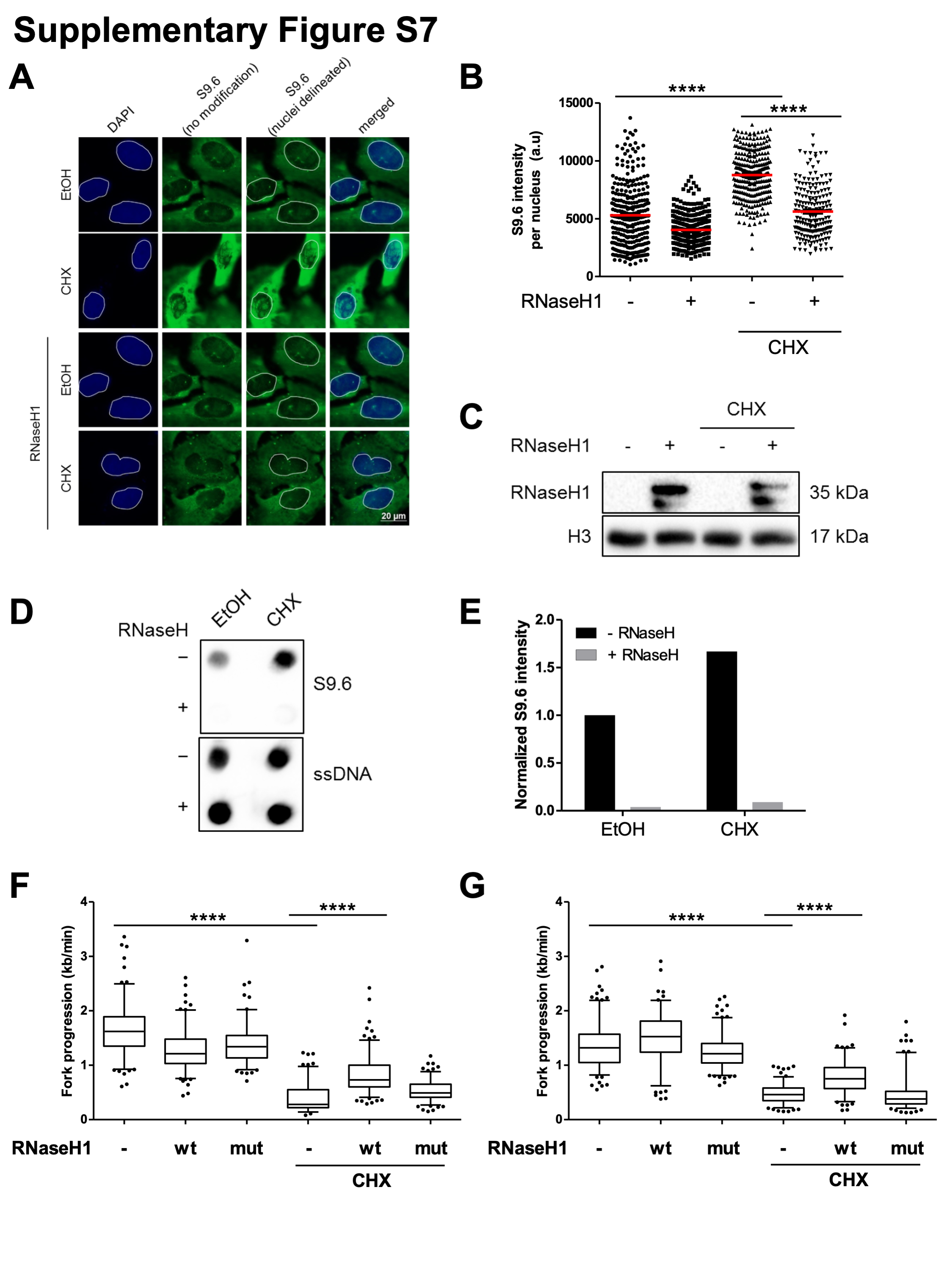

Supplement: Supplementary file 7 — Supplementary figure 7 [file 41419_2020_2727_MOESM7_ESM.tif]
