# Supplementary material for: The integrated stress response induces R-loops and hinders replication fork progression
Source: Cell Death Dis. 2020 Jul 16;11(7):538. doi: 10.1038/s41419-020-2727-2 (PMC7366693; doi:10.1038/s41419-020-2727-2)
Supplement: Supplementary file 11 — Supplementary table legends [file 41419_2020_2727_MOESM11_ESM.docx]

**The integrated stress response induces R-loops**

**and hinders replication fork progression**

Josephine Ann Mun Yee Choo ^a^, Denise Schlösser ^a^, Valentina Manzini ^a^, Anna Magerhans ^a^ and Matthias Dobbelstein ^a,1^

1. *Institute of Molecular Oncology, Göttingen Center of Molecular Biosciences (GZMB), University Medical Center Göttingen, D-37077 Göttingen, Germany*
2. *Corresponding author. Correspondence and requests for materials should be addressed to M. D. (phone: +49 551 39 60757; fax: +49 551 39 60747; e-mail:* [*mdobbel@uni-goettingen.de*](mailto:mdobbel@uni-goettingen.de)

Running title: ISR antagonizes DNA replication

Keywords: Integrated stress response, PKR, PERK, GCN2, eIF2alpha, Thapsigargin, BEPP, ISRIB, R-loops, histones, DNA replication, DNA fiber assays

Declaration of interests: The authors declare no conflict of interests

**SUPPLEMENTARY TABLE LEGENDS**

**SUPP TABLE 1: Fiber assay summary**

Table summarizing the mean, median, standard deviation and number of labeled tracks sampled for each condition of the fiber assay experiments shown as part of the main figures.

**SUPP TABLE 2: Fiber assay raw data**

Raw values of track lengths (both CldU and IdU) in each fiber measured using the Fiji software. As described in the methods and materials section, these fiber lengths (in pixels) were converted into micrometers and subsequently into kilobases prior to obtaining the final readout in terms of kilobases per minute. Each dataset (fork progression of each fiber measured) was then collectively plotted and represented as box plots shown in the figure panels. Only the IdU fork progression for each condition is shown in the figures.
